# Genetic dosage and position effect of small supernumerary marker chromosome (sSMC) in human sperm nuclei in infertile male patient

**DOI:** 10.1038/srep17408

**Published:** 2015-11-30

**Authors:** Marta Olszewska, Elzbieta Wanowska, Archana Kishore, Nataliya Huleyuk, Andrew P. Georgiadis, Alexander N. Yatsenko, Mariya Mikula, Danuta Zastavna, Ewa Wiland, Maciej Kurpisz

**Affiliations:** 1 Institute of Human Genetics, Polish Academy of Sciences, Department of Reproductive Biology and Stem Cells, Strzeszynska 32, 60-479 Poznan, Poland; 2 Department of Obstetrics, Gynecology and Reproductive Sciences, University of Pittsburgh School of Medicine, Pittsburgh 15213, PA, USA; 3 Institute of Hereditary Pathology, Ukrainian Academy of Medical Sciences, Lysenko Str. 31a, 79000 Lviv, Ukraine

## Abstract

Chromosomes occupy specific distinct areas in the nucleus of the sperm cell that may be altered in males with disrupted spermatogenesis. Here, we present alterations in the positioning of the human chromosomes 15, 18, X and Y between spermatozoa with the small supernumerary marker chromosome (sSMC; sSMC^+^) and spermatozoa with normal chromosome complement (sSMC^−^), for the first time described in the same ejaculate of an infertile, phenotypically normal male patient. Using classical and confocal fluorescent microscopy, the nuclear colocalization of chromosomes 15 and sSMC was analyzed. The molecular cytogenetic characteristics of sSMC delineated the karyotype as 47,XY,+der(15)(pter->p11.2::q11.1->q11.2::p11.2->pter)mat. Analysis of meiotic segregation showed a 1:1 ratio of sSMC^+^ to sSMC^−^ spermatozoa, while evaluation of sperm aneuploidy status indicated an increased level of chromosome 13, 18, 21 and 22 disomy, up to 7 × (2.7 − 15.1). Sperm chromatin integrity assessment did not reveal any increase in deprotamination in the patient’s sperm chromatin. Importantly, we found significant repositioning of chromosomes X and Y towards the nuclear periphery, where both chromosomes were localized in close proximity to the sSMC. This suggests the possible influence of sSMC/XY colocalization on meiotic chromosome division, resulting in abnormal chromosome segregation, and leading to male infertility in the patient.

Supernumerary marker chromosomes (sSMC) are small, structurally abnormal chromosomes that occur in addition to the normal set of 46 chromosomes. Overall, 75% of sSMCs are *de novo*^1^. Most cases of sSMC are developmentally normal (about 74% of *de novo* sSMC cases and over 98% of parentally transmitted cases). The sSMC frequency in newborns is 0.044% (0–0.219%), while in patients with fertility problems, the sSMC rate increases to 0.125%^2^. sSMC frequency is also 7.5-fold higher in male carriers (0.165%) than in females (0.022%)^3^. It is estimated that approx. 30% of sSMCs are derived from chromosome 15^2^,^4^. It has also been observed that maternally inherited sSMCs are ~1.6-fold more frequent than paternally inherited sSMCs (16% vs. 7.0%) and are more likely to be observed in a son with fertility problems that in daughter^2^,^5^.

In infertile carriers, up to 85% of sSMCs originate from acrocentric chromosomes, mostly from chromosome 15 (approximately 45%), and in more than 50% of cases, the sSMCs are parentally inherited^2^. To this day, the role of sSMCs in reproduction is not clearly understood, though some observations suggest that they have an influence on fertility status^2^,^6^. Statistics have shown that, in cases of unexplained infertility and of repeated spontaneous abortions, the frequency of sSMC is increased (22–47%)^2^. In males with decreased sperm parameters (oligozoospermia or oligoasthenoteratozoospermia) sSMC incidence is also remarkably elevated (7%)^2^. Such disturbances may result not only from selection mechanisms against additional chromosome content during spermatogenesis, leading to a decrease in the number of gametes, but also the incidence of sister chromosomes (demonstrated for *de novo* sSMCs and in cases with repeated spontaneous abortions), and from some unknown epigenetic factors.

It is known, that in the nucleus of diploid cells, chromosomes are localized nonrandomly in chromosome territories (CT). Together with interchromatin compartments (ICs) and other elements of the nuclear matrix, CTs form the so-called intranuclear architecture^7^,^8^. The size and localization of the CTs depend on the size of the chromosomes, gene density, transcriptional activity, cell-cycle stage, and cell type. There are strong suggestions that proper spatial organization of the genome may create an important epigenetic layer of cellular control mechanisms^9^,^10^,^11^. When compared to somatic cell types, in human spermatozoa the condensation of sperm chromatin and CTs is 4–6 time stronger and is triggered by the exchange of histones to protamines^10^,^11^,^12^,^13^. Sperm chromosomes are looped into a hairpin structure with their centromeres directed toward the nucleus center (the chromocenters), while the telomeres show a tendency to occupy the nuclear periphery where they form dimers and tetramers^10^,^14^,^15^. It has been suggested that telomeres are the first element of the paternal genome to directly contact the ooplasm after fertilization. Such a chromosome conformation seems to be required for normal fertilization and zygote development^16^,^17^,^18^. Data from several studies have clearly shown that the chromosomes in human spermatozoa also occupy well-defined settings^11^,^19^,^20^,^21^. Chromosome positioning is determined during the meiotic stages of spermatogenesis^11^,^22^. Taking different criteria of sperm nucleus division models, nonrandom positions of all the chromosomes in human sperm nuclei have been presented so far^11^,^16^,^19^,^21^,^23^,^24^,^25^. It has also been shown that, in spermatozoa, there is a possible association between chromosome position, its size, and its gene density, as in diploid cells^11^,^20^. Data obtained from previous studies indicate that the nuclear order of chromosomes can be altered in males with disturbed spermatogenesis, particularly observed in males with increased sperm aneuploidies, reciprocal translocation carriers, and decreased semen parameters^23^,^24^,^25^,^26^,^27^. Furthermore, one study described the colocalization of sSMC with its sister chromosome in spermatozoa from the ejaculates of two brothers^4^.

In the present study, we aimed to analyze the differences in chromosome topology in spermatozoa with (sSMC^+^) and without (sSMC^−^) the marker chromosome obtained from the same ejaculate of the sSMC carrier. We therefore compared the spatial localization of the centromeres of chromosomes 15, 18, X and Y in sSMC^+^ vs. sSMC^−^ spermatozoa, also including the positioning of the marker chromosome. Moreover, molecular and cytogenetic methods were used to ascertain the karyotype of the carrier, followed by evaluations of meiotic segregation, sperm aneuploidy level, and chromatin deprotamination status.

## Results

### Characterization of sSMC

**Figure 1:**
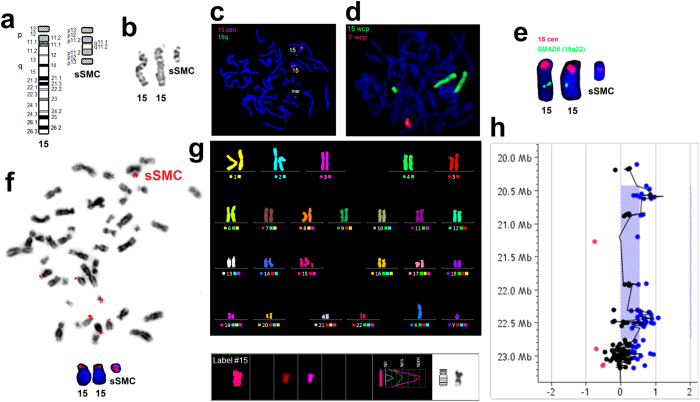
Chromosome staining results: characteristics of the sSMC. (**a**) Ideograms of chromosome 15 and the observed sSMC. (**b**) GTG banding. (**c**) FISH with probes for chromosome 15: centromere-specific (red) and subtelomeric (green), pointing to a lack of subtelomere region in the sSMC. (**d**) FISH with whole chromosome painting probes for chromosomes: 15 (green) and Y (red) (**e**) FISH with centromere-specific probe for chromosome 15 (red) and gene-specific probe for *SMAD6* (15q22.31; green). (**f**) Acro-p FISH result showing two NOR regions on both ends of the sSMC. Displaying modes: inverted DAPI with the whole view of the metaphase plate and DAPI with close-up of chromosomes 15 and sSMC. (**g**) mFISH analysis showing that sSMC was structured from only chromosome 15 material. (**h**) aCGH image showing a 15q11.1-q11.2 gain of ~2323 Kb size with a log ratio of 0.547, indicating a one-copy amplification in the region, which indicates the content of the sSMC.

The characteristics of the analyzed sSMC are presented in Fig. 1. The use of wcp-FISH on the metaphase lymphocytes identified sSMC as being derived from chromosome 15 in 100% of the tested cells. mFISH analysis excluded the addition of any other chromosomal component to sSMC. FISH using centromeric probe for chromosome 15 and subtelomeric probe for 15q showed no subtelomere 15q presence in sSMC and at least a twice as small size for the sSMC centromere when compared to the centromere of chromosome 15. The presence of a small slice of centromeric region found in sSMC, was then confirmed by aCGH. Acro-p FISH showed the presence of nucleolar organizing regions at two ends of the analyzed sSMC. To confirm the FISH findings and to identify the size of the sSMC material, we performed a 400 K aCGH experiment. The aCGH analysis resulted in 5 copy number variations (CNVs) consisting of one small deletion and 4 amplifications (see Supplementary Fig. S1, and Supplementary Tab. S2 online). Three amplifications and a deletion had already been reported as polymorphic genomic variants in the DGV database, and one small amplification involving *C7orf50* was intronic. They were thus not considered pathogenic. One large genomic region of ~2323 kb on chromosome 15 showed a gain with a log ratio of 0.547, indicating an extra copy gain in the 15q11.1-q11.2 region. The DGV database shows smaller polymorphic gains and losses in this region, but none comparable to this detected one, suggesting that this region is included in the sSMC genomic material. The amplified region includes 15 known genes based on human genome build hg19, *HERC2P3, GOLGA6L6, GOLGA8C, BCL8, POTEB, NF1P1, LOC646214, CXADRP2, LOC727924, OR4M2, OR4N4, OR4N3P, REREP3, GOLGA8DP, GOLGA6L1* (see Supplementary Fig. S1 and Supplementary Tab. S3 online). The karyotype of the sSMC carrier based on FISH and CGH is: 47,XY,+der(15)(pter->p11.2::q11.1->q11.2::p11.2->pter)mat.ish der(15)(wcp15+,D15Z4+,acro-pNOR++,qter−,*SMAD6*−).arr[hg19] 15q11.1q11.2(20,432,851–22,756,709)×3.

### Sperm chromatin deprotamination

The analysis of sperm chromatin in the sSMC carrier showed 19.41% (AB staining) and 20.07% (CMA3) spermatozoa with deprotaminated chromatin. No statistical significance (P > 0.05) was observed when the patient’s results were compared to the mean control values (AB: mean 16.53 ± 7.89%, range: 6.2–32.3%; CMA3: mean 21.79 ± 7.79%, range: 8.57–31.85%).

### Meiotic segregation and aneuploidy level

We analyzed the meiotic segregation of sSMC and the aneuploidy status in the spermatozoa using two- or three-color FISH. The frequencies of common genotypes are presented in Table 1. Two rounds of FISH staining with wcp probes allowed chromosome 15 to be differentiated from sSMC through their distinct sizes and a lack of *SMAD6* gene signal. Thus, the mean frequency of spermatozoa without sSMC was estimated at 47.58%, while the frequency with one chromosome 15 and one sSMC as 51.20%. Further staining with centromere-specific probe for chromosome 15 allowed the estimation of frequencies for spermatozoa with the two 15cen signal (15, 15 or 15,sSMC). The frequencies obtained were similar to the wcp results (Table 1), so we assumed that the ratio sSMC^−^:sSMC^+^ was 1:1. The ratio of X:Y chromosomes was estimated at 1.02.

Aneuploidy analysis for chromosomes 21, 22, X and Y showed that there were no differences (P > 0.05) between sSMC^+^ and sSMC^−^ gametes, while for chromosomes 13 and 18, the frequency of disomic sSMC^+^ spermatozoa was twice as high (13) or twice as low (18) (P < 0.05) than disomic sSMC^−^ (Table 1). When the disomy frequencies of sSMC carriers’ spermatozoa were juxtaposed against control results, statistically significant differences were found as follows: (i) the frequencies of all the evaluated autosomal (13, 18, 21, 22) disomies were significantly higher (P < 0.05) than the mean control rates of fertile healthy males, while for the sex chromosomes, the observed frequencies were similar (P > 0.05; sSMC^+^) or lower (P < 0.05; sSMC^−^) (Table 1); (ii) when compared to the results of the RF-group, the frequencies of all gonosomal disomies (XX, XY, YY), as well as of chromosome 21, were lower (P < 0.05), while those of chromosomes 13 and 18 were higher in sSMC carrier (P < 0.05) (Table 1). When collating mean values of two control groups (control vs. RF-control), the disomy frequencies of all analyzed chromosomes were significantly higher in RF control group (P < 0.05) (Table 1).

### Topology of chromosomes

**Figure 2:**
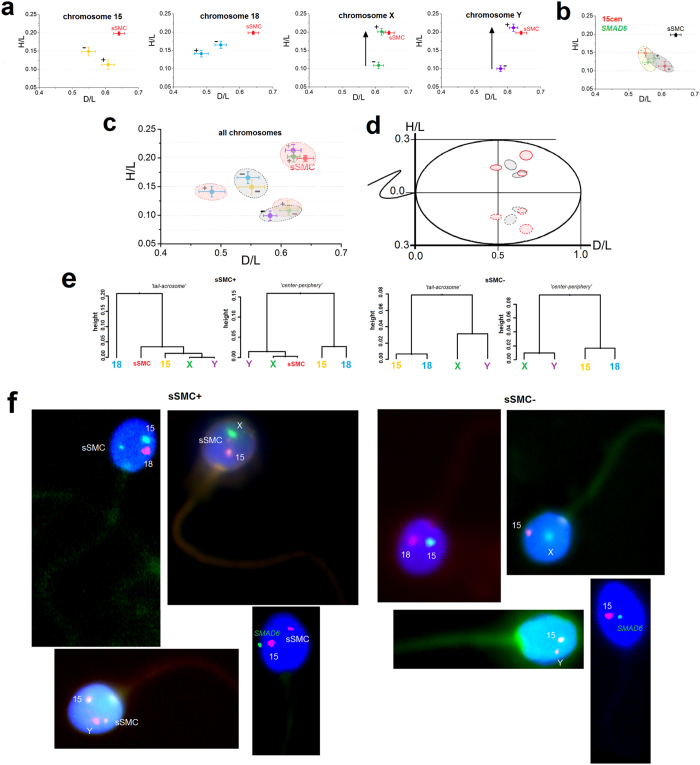
Schematic representation of the radial analysis of the localization of chromosome 15, 18, X, Y and sSMC centromeres, according to the results from Table 2. (**a**) Comparison of the centromere positioning of individual chromosomes in sSMC^+^ and sSMC^−^ spermatozoa. The arrows indicate the directions of statistically significant shifts of the centromeres. (**b**) Comparison of the suggested chromosome 15 territory localization according to *SMAD6* (15q22.31) gene positioning (grey color for sSMC^+^ spermatozoa and yellow for sSMC^−^). (**c**) Spatial nuclear area encompassing the positions of all the evaluated chromosomes in fragments of chromocenter(s) in sSMC^+^ gametes (red areas) and sSMC^−^ spermatozoa (grey areas). (**d**) Fragments of possible chromocenter(s) with a perspective of the whole sperm nucleus. Solid lines represent the observed areas, while dotted lines show their mirror-images. (**e**) Dendrogram classification of centromere clusters in sSMC^+^ and sSMC^−^ spermatozoa according to both positioning criteria. In the sSMC^+^ gametes, chromosomes X and Y were clustered together with the sSMC, while in the sSMC^−^ gametes there were two groups of chromosomes: 15/18 and X/Y, as in (**c**) part of the figure. (**f**) Examples of sperm FISH phenotypes following hybridization with centromere-specific probes for chromosomes 15, 18, X and Y and a gene-specific probe for *SMAD6*.

The determined radial positioning (2D) of the centromeres of chromosomes 15, 18, X, Y and sSMC in the sperm cell nucleus are shown in Table 2 and Fig. 2. When comparing sSMC^+^ and sSMC^−^ spermatozoa, statistically significant differences (P < 0.01) were found only in the case of sex chromosomes, according to the criterion of the nucleus depth (‘center-periphery’; H/L values). It was found that, in sSMC^+^ gametes, the X and Y centromeres were strongly repositioned towards the nucleus periphery (X: H/L = 0.201; Y: H/L = 0.212) when collating with sSMC^−^ spermatozoa (X: H/L = 0.109; Y: H/L = 0.100) (X and Y: P < 0.0001) (Fig. 2a). Moreover, when collating the localization of sSMC vs. the sex chromosomes in sSMC^+^ gametes, similar positioning was noted (P > 0.01) (Fig. 2a). In case of chromosome 15 vs. sSMC, it was found that, in sSMC^+^ gametes, centromere 15 was localized deep in the nucleus of the spermatozoa, while sSMC had a position near the nuclear periphery (P < 0.0001) (Table 2, Fig. 2a). No statistical differences (P > 0.01) in centromere positioning were observed for chromosomes 15 and 18 when collating sSMC^+^ vs. sSMC^−^ spermatozoa (Fig. 2a). Similarly, the results of positioning the *SMAD6* locus (15q22.31) show that the topology of the gene had unaltered positions (P > 0.01) both in sSMC^+^ (D/L = 0.575; H/L = 0.134) and sSMC^−^ (D/L = 0.557; H/L = 0.123) spermatozoa (Table 2, Fig. 2b).

In sSMC^+^ spermatozoa, the centromeres were more dispersed than in the case of sSMC^−^ gametes (Fig. 2c,d). Hierarchal Ward cluster analysis showed that in sSMC^+^ spermatozoa, chromosomes X and Y were aggregated together with sSMC, while in sSMC^−^ gametes, two clusters of chromosomes were observed: 15 + 18 and X + Y (Fig. 2e).

The normalized distance measurements between the centromeres of normal chromosome 15 vs. sSMC revealed similar values in spermatozoa bearing X or Y chromosomes (X: 1.480 ± 0.662 μm; Y: 1.464 ± 0.672 μm; P = 0.8655). No differences between X-bearing and Y-bearing gametes were observed between the centromeres of sSMC and the X/Y centromeres: sSMC-X 1.835 ± 0.682 μm vs. sSMC-Y 1.679 ± 0.685 μm (P = 0.1081). Statistically significant differences were found between the sSMC^+^ and sSMC^−^ spermatozoa when comparing distances between chromosome 15 and sex chromosomes: 15-X (sSMC^+^) 2.003 ± 0.789 μm vs. 15-X (sSMC^−^) 1.783 ± 0.523 μm (P = 0.0211) and 15-Y (sSMC^+^) 1.858 ± 0.789 μm vs. 15-Y (sSMC^−^) 1.505 ± 0.650 μm (P = 0.0007). No statistical differences were noted when comparing the distances in sSMC^+^ spermatozoa for 15-X vs. sSMC/X (P = 0.1088) and 15-Y vs. sSMC/Y (P = 0.0883).

**Figure 3:**
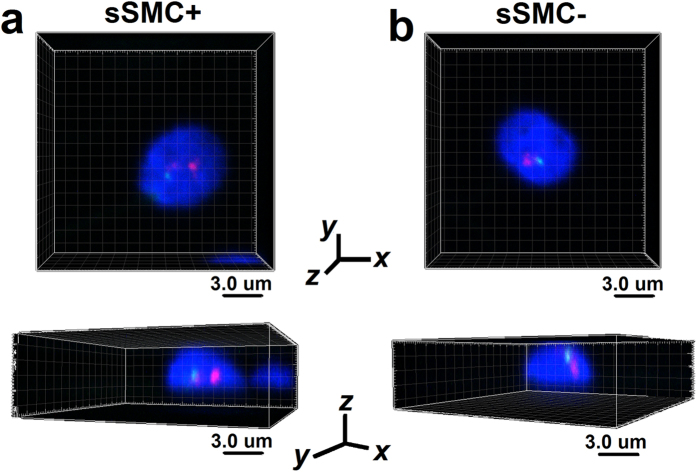
The confocal representation of the positioning of sSMC and chromosome 15 centromeres demonstrated in two geometrical perspectives obtained by confocal microscopy. Red: centromere-specific probe for chromosome 15; green: *SMAD6* gene *locus* (15q22.31). Scale bar: 3.0 μm. (**a**) Spermatozoa bearing sSMC (sSMC^+^) with two red and one green FISH signals. (**b**) Spermatozoa without sSMC (sSMC^−^) with one red and one green FISH signals.

The positioning results for the sSMC and chromosome 15 centromere using confocal analysis (3D) corresponded with the radial results. Representative imagery from confocal microscopy are presented in Fig. 3 and the Supplementary files, which contain animations of spermatozoa with and without sSMC (see Supplementary Videos S4 and S5 online). There was a statistically significant difference (P < 0.0001) in the frequencies of centromere 15’s localization in particular areas between the sSMC^+^ and sSMC^−^ spermatozoa. In the sSMC^+^ sperm, the nucleus of almost all (92.5%) of the centromeres of chromosome 15 were localized centrally (shell no. 1 ‘cen’), followed by a small frequency in shell no. 2 (‘int’ 7.5%), and a lack of signals in a shell no. 3 (‘per’ 0%) (counted according to description in ‘Materials and Methods’). In the sSMC^−^ spermatozoa, the highest frequency of chromosome 15 centromeres (87.5%) was noted for the ‘int’ shell no. 2, while for the remaining shells, the frequencies were lower (no. 1 ‘cen’ 12.5%; no. 3 ‘per’ 0%). sSMC strongly preferred the intermediate ‘int’ position (shell no. 2, 97.5%) (shell no. 1 ‘cen’ 2.5%; no. 3 ‘per’ 0%).

All these three-point positioning measurement approaches (radial, distances and confocal) complement one another and give a broad insight into the topology of chromosomes in spermatozoa.

## Discussion

In this study, a series of experiments showed that maternally inherited sSMC was composed of two p-arms and fragment of chromosome 15 (q11.1->q11.2). The carrier’s karyotype was established as 47,XY,+der(15)(pter->p11.2::q11.1->q11.2::p11.2->pter)mat. The most probable model for the formation of the observed sSMC is an intrachromosomal or interchromosomal U-type exchange between low-copy repeats (LCRs) of homologous chromosomes, as a result of a cross-over error during meiosis^28^. It should be noticed that region 15q11.2-12 is regarded as one of the points in the genome that is most prone to chromosomal breakpoints leading to different types of genomic rearrangements (duplications, deletions, insertions, translocations, etc.)^29^,^30^. The molecular analysis performed on the sSMC showed an euchromatic region (~2323 kb in size) with polymorphic genomic variants of 15 genes that were not considered to be pathogenic in the case of the patient, who was phenotypically normal. This finding seems to confirm previous data from the literature indicating a non-dose-sensitive region of chromosome 15 as containing an entire short arm, followed by a lack of euchromatin or only its small proximal part^31^,^32^.

So far, meiotic segregation of the marker chromosomes in sperm has been examined in only twelve cases, including six carriers of sSMC(15), one of sSMC(14), one of sSMC(20), two of sSMC(22), and two of unknown origin^6^,^33^,^34^,^35^,^36^,^37^,^38^,^39^,^40^. The frequency of spermatozoa bearing sSMC in non-mosaic cases (n = 10) ranged from 11.5 to 51%. Our case, where 50.76% of spermatozoa were sSMC^+^, is consistent with two studies^38^,^39^ that showed the expected 1:1 segregation ratio in the spermatozoa of three infertile sSMC carriers. The lower sSMC frequencies obtained by other authors (6.2–37%) suggest the existence of some selection mechanism against sSMC during spermatogenesis or a tissue-specific mosaicism that resulted from the instability of the sSMC, its decreased replication success, the slower cell cycle of sSMC-bearing cells, or a random loss of the sSMC during successive mitotic divisions^6^,^33^,^34^,^36^,^37^,^40^. The spermiogram of the carrier in a recent study showed OAT (oligoasthenoteratozoospermia) and seems to also confirm previous findings of the higher incidence of sSMC in males with decreased seminology^2^,^6^,^35^,^38^,^40^. Disruption of spermatogenesis may also occur when the sSMC associates with the bivalent XY, which may lead to a drop in semen parameters^40^,^41^,^42^. Kirkpatrick *et al.*^40^ observed that sperm concentration was 10× decreased in a carrier of 46,XY,rob(13;21),+mar when compared to a 45,XY,rob(13;21) without the marker chromosome. Additionally, when collating decreased seminological quality in our sSMC carrier (=abnormal karyotype) vs. normozoospermia in RF-control males (=normal karyotype), this seems to pose a question about the possible influence of sSMC on seminal parameters. However, the presence of sSMC on its own does not necessarily imply lower seminological parameters or infertility, as it was previously documented by the observation of normozoospermic sSMC carriers^34^, fertile carriers with miscarriages in partners^2^, and brothers with different (in)fertility histories^6^. It is also known that the presence of sSMC does not reduce the fertilizing potential of spermatozoa, what has been clearly documented using human–hamster sperm penetration assay (SPA) in sSMC carriers with unexplained infertility^43^,^44^. Thus, we cannot exclude the possibility that in the present study sSMC carrier had spermatozoa that were able to fertilize; unfortunately, it was not possible to determine this via SPA. Additionally, we cannot exclude the possibility of unrecorded early miscarriages in carrier’s spouse, whose documented fertility (a daughter with another man) may suggest that the reason for the failures lay on the male side. Some indirect role of sSMC in male infertility cannot be entirely excluded, because of the fact that sSMCs that are predominantly inherited maternally (mat vs. pat 1.8–2:1) lead to infertile male offspring^1^,^2^,^5^,^37^. Up to 30% of infertile male sSMC carriers present unexplained infertility^2^. It is also known that in phenotypically normal but infertile males the frequency of marker chromosomes is much higher than in the normal population (0.125% vs. 0.044%)^1^.

In our study, we also observed an approximately 7 times (2.7 − 15.1×) higher level of autosomal disomy in the sSMC carrier’s spermatozoa, suggesting the incidence of interchromosomal effect (ICE). It is well known that increased aneuploidy levels may result in reproductive failures or a decrease in fertility^45^,^46^,^47^. Sperm aneuploidies originate from meiotic errors induced by constitutional genetic abnormalities (impaired synapsis, decreased recombination rate, association with XY bivalent) or an altered testicular environment^47^. The aneuploidy level is commonly being estimated for chromosomes 13, 15, 16, 18, 21 and 22. Trisomies of chromosomes 13, 18 and 21 are being observed in trisomic live births and are responsible for congenital malformations, whereas trisomies of chromosome 15, 16 and 22 are linked to a higher risk of miscarriages^48^. Sperm aneuploidy was estimated in ten sSMC cases, so far, including only four men with sSMC(15)^6^,^33^,^34^,^36^,^37^,^39^,^40^. In published data concerning aneuploidies in sSMC(15) carriers, the disomy level did not differ from the control value in only one study^37^. Oracova *et al.*^34^ included also blastomeres testing in PGD (prenatal genetic diagnosis)^34^. Authors performed aneuploidy screening for chromosomes 13, 15, 16, 18, 21, 22, X and Y, and have found that in sperm the disomy level was elevated for chromosomes 21 and 22^34^. It should be noticed that the visible problem of aneuploidies in the sperm of sSMC carriers may suggest that, as with carriers of other structural aberrations, sSMC carriers are more prone to exhibiting higher aneuploidy incidents, especially with regard to the known possible influence of chromosomal rearrangements on the proper meiotic behavior of other chromosomes^47^. It has been documented that increased aneuploidy rates can be found in the spermatozoa of males with decreased sperm seminological parameters (OAT), and especially those with oligozoospermia^20^,^21^,^46^,^49^. We cannot avoid the suggestion that, in the evaluated sSMC case, both the presence of SMC, and the higher disomy level of the autosomes may influence the meiotic process leading to disrupted spermatogenesis, which is reflected as OAT on the seminogram.

In this report, we applied a chromosome topology analysis in spermatozoa derived for the first time from the same ejaculate of an sSMC infertile carrier. The comparison of the spatial position of centromeres of chromosomes 15, 18, X and Y was performed in sperm nuclei with (sSMC^+^) and without (sSMC^−^) the marker chromosome. We observed that in sSMC^+^ spermatozoa, the centromeres of the investigated chromosomes were more dispersed within the nuclear space (three fragments of the chromocenter vs. two in the sSMC^−^ gamete; Fig. 2d). This higher dispersion of the chromosomes was previously also reported in patients with other pathologies of the chromosomes, such as reciprocal translocations^23^, and in males with a higher rate of sperm aneuploidies^25^,^27^. The alterations of the nuclear order of chromosomes 18, X and Y were documented in the spermatozoa of patients with isolated teratozoospermia^50^. The authors suggested that the disturbed chromosome localization, as a representation of chromatin integrity, was clearly linked to nuclear vacuoles^50^. However, decreased seminological parameters (OAT) by themselves do not guarantee the disruption of nuclear organization^24^,^25^,^51^.

The positioning of sSMC has been described previously in a study of spermatozoa^52^ and in another describing its localization in somatic cells^4^. In both reports, it was found that sSMCs were localized near to their sister chromosomes. In our study, distance measurements confirm the colocalization of sSMC and chromosome 15. Moreover, we did not find any repositioning of the chromosome 15 centromere. Karamysheva *et al.*^52^ have found that in the spermatozoa of an infertile brother, sSMC(15) preferred colocalization with sex chromosomes more frequently than in the fertile brother. A similar finding was noted in our study with the repositioning of the sex chromosomes towards the periphery of the cell nucleus in sSMC^+^ gametes, resulting in colocalization to the sSMC (Fig. 2). It is well known that in normal fertile human spermatozoa, chromosomes X and Y prefer positioning deep in the nucleus center^11^,^23^,^24^,^25^,^27^,^53^. There are also strong suggestions that such positioning is crucial for the proper reorganization of the paternal genome after fertilization^15^,^16^,^18^. The essential visible alterations of the sex chromosomes were also found in the spermatozoa of infertile males with higher rates of sperm aneuploidies^25^,^27^, in reciprocal translocation carriers^23^, and in an infertile brother who carried of sSMC^52^. We fully agree with the two studies suggesting a possible linkage between ICE in sSMC carriers and the nuclear positioning of gonosomes^40^,^52^. All those data imply the possible effect of any chromosomal aberration via disturbances in meiosis, on the proper nuclear order of the spermatozoa. Thus, concerning the case evaluated in the present study, two hypothesis can be suggested: (i) in some of the spermatogenic cells the colocalization of sSMC and bivalent XY leads to their association in pachytene, decreasing the number of cells that can pass on to the next meiotic steps, leading in consequence to a decrease in the total sperm number in the ejaculate; (ii) the close positioning of sSMC and X or Y may also influence the first mitotic divisions in the zygote, leading to an inhibition of further development and, as a result, to unrecorded early miscarriages in the carrier’s wife. Those suggestions may be supported by the fact that in the pachytene spermatocytes, the preferential proximity between bivalent XY and the acrocentrics (especially chromosomes 15 and 22) has been well documented both in fertile men^54^,^55^ and in infertile males with different etiologies of infertility^51^,^56^. What is interesting, an association of sSMC(22) and XY has been found by Kirkpatrick *et al.*^40^, in 50% of sSMC^+^ pachytene spermatocytes of a 47,XY,+der(22) carrier. The extremely close localization of about 30–40% of chromosome 15 bivalents to XY may be explained by the high homology found between regions of these chromosomes, such as: noncentromeric heterochromatin fragments of chromosome 15 and a part of the Xq/Yq subtelomeric sequences^54^,^55^. We can therefore speculate that the observed repositioning of chromosomes X and Y towards the nuclear periphery (where both are localized closer to the sSMC than to chromosome 15) may originate from meiotic disturbances.

Thus, we can conclude that, in the case of sSMC here considered, the presence of the marker chromosome may be not neutral for the the chromosome topology in the sperm nuclear space, leading in consequence to infertility of studied individual.

## Materials and Methods

### Patient

**Figure 4:**
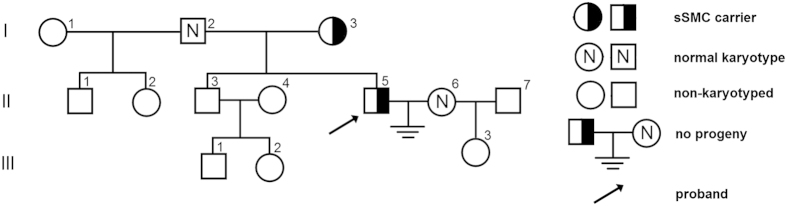
The family pedigree of the infertile sSMC carrier (II:5) karyotyped as 47,XY,+der(15)(pter->p11.2::q11.1->q11.2::p11.2->pter)mat.

The family pedigree of the investigated sSMC case is shown in Fig. 4. The material for the analyses consisted of peripheral blood lymphocytes and spermatozoa from a 30-year old carrier (II:5) of marker chromosome 47,XY,+mar inherited maternally (I:3). The spermiogram exhibited decreased concentration, morphology and motility (moderate oligoasthenoteratozoospermia, OAT; with sperm concentration 10 × 10^6^/ml) (according to WHO, 2010^57^). The carrier presented a lack of conception over a 7-year period, though his wife (II:6) had normal karyotype and a healthy daughter (III:3) with another partner (II:7).

### Control groups

Our laboratory control material consisted of sperm cells from healthy, fertile male donors (n = 7 or 15, depending on the analysis) aged between 25 and 30 with normozoospermia (according to WHO criteria^57^). Additionally, for aneuploidy evaluation, we employed a second comparison group consisting of 7 normozoospermic males with normal karyotypes but with documented reproduction failures, hereafter referred as the ‘RF-group’. We decided to use this group to check, if the presence of sSMC on its own may influence the seminal parameters via disruption of meiotic process. Ejaculated sperm samples from all men were collected after 3–5 days of sexual abstinence. After liquefaction and washing in F-10 medium, the sperm samples were fixed with a fresh fixative solution (methanol:acetic acid, 3:1 v/v, −20 °C) and stored at −20 °C until further use. All males (proband and controls) were notified of the purpose of the research, in line with the guidelines of the Local Bioethical Committee, Poznan University of Medical Sciences, and written informed consent was obtained from all the subjects. All the procedures performed within our project were conducted in the accordance with the approved by Committee protocols.

### Characterization of sSMC in peripheral blood lymphocytes

Classical karyotyping using Giemsa staining (GTG bands on peripheral blood lymphocytes at metaphase stage, fixed in freshly made solution of methanol:acetic acid 3:1 v/v, −20 °C) showed sSMC presence in all (n = 30) spreads examined under the light microscope (Zeiss D1 AxioImager). For identification and characterization of the evaluated sSMC, fluorescence *in situ* hybridization (FISH) and array CGH (aCGH) experiments were prepared.

#### FISH on peripheral blood lymphocytes

The following combinations of FISH probes were used:

- whole chromosome painting probes (wcp) for chromosomes 15 (green) and Y (red) (MetaSystems, Germany), each 8.0 μl;
- α-satellite (centromere-specific) probes for chromosome 15 (*locus* D15Z4; green, 2.5 μl) and subtelomeric for 15q (clone 154P1; red, 3.0 μl) (Cytocell, UK), filled with hybridization solution (HS) to a final volume of 10.0 μl;
- α-satellite for chromosome 15 (*locus* D15Z4; red, 1.5 μl; Cytocell, UK) and *SMAD6* gene-specific probe (*locus* 15q22.31; green, 0.5 μl; Agilent, USA), filled with HS to 5.0 μl;
- acro-p for NOR regions in acrocentric chromosomes (Cytocell, UK), 10.0 μl;
- mFISH (multicolor FISH) (MetaSystems, Germany), 10.0 μl.

#### Microscopy and data analysis

FISH results were scored using a fluorescent microscope (Zeiss D1 AxioImager) with an oil immersed objective 100× and a proper filter set (FITC/Texas Red/SpO/Cy5/DEAC/DAPI). Images were acquired with a CCD camera and analyzed using Ikaros/ISIS software (MetaSystems, Germany). Detailed observation of the sSMC was performed for 30 metaphase spreads in each FISH staining combination. Additionally, for each FISH combination, at least 1,000 interphase lymphocytes were counted to determine whether sSMC was present in 100% of the cells. The efficiency of FISH was estimated at 99%.

#### aCGH analysis

To test for potential genomic micro-aberrations and describe the detailed nature of genomic region amplification in the chromosome 15 involved in the sSMC, we performed a genome-wide analysis using the SurePrint G3 Human CGH 2 × 400 k Oligo Microarrays (Agilent Technologies, Santa Clara, CA, USA). The analysis was performed according to the manufacturer’s protocol (Agilent Technologies, Santa Clara, CA), as described previously^58^. Briefly, genomic DNA was extracted from mother’s peripheral blood leukocytes (Qiamp MiniKit; Qiagen). Reference female DNA was purchased from Promega (Madison, WI). DNA was labeled with Cyanine-5 dye, while the reference DNA was labeled with Cyanine-3 dye. The labeled DNA was hybridized to the probes for 40 hours at 66 °C. After washing, the slides were scanned on an Agilent SureScan Microarray C scanner and analyzed using Agilent CytoGenomics software. The quality of the DNA gains and losses of all variants was assessed using the direct probe signal intensity and the log^2^ ratio of the patient:reference signals. The resultant CNVs were checked against the DGV database (Toronto CNV database) to verify the frequency of these CNVs in the normal population.

### Analysis of spermatozoa

#### Sperm chromatin deprotamination

The status of chromatin maturity was determined using two described previously staining methods^59^. The first method, aniline blue staining (AB; Water Blue, Fluka, Germany), relies on the binding of aniline to the lysine-rich residues of histones, resulting in a dark blue color of the sperm chromatin. The second method, chromomycin A3 (CMA3; Sigma-Aldrich), is a fluorescent indicator of a protamine-free GC sites of DNA. For the sSMC carrier, 5,460 spermatozoa were analyzed for AB and 1,166 for CMA3 stainings. The control results consisted of our laboratory group (n = 15) with at least 5,000 spermatozoa analyzed for AB and 1,000 for CMA3 in each control male.

#### FISH on spermatozoa

The following combinations of FISH probes were used for meiotic segregation of sSMC, sperm aneuploidy rates (for chromosomes 13, 15, 18, 21, 22, X and Y), and topology in the sperm cell nucleus (chromosomes 15, 18, X, Y and sSMC):

- wcp for chromosomes 13 (red; 7.0 μl) and 15 (green; 7.0 μl ; MetaSystems, Germany) and α-satellite for chromosome 18 (*locus* D18Z1; blue; 3.0 μl) (Cytocell, UK), filled with HS to a final volume of 20.0 μl;
- wcp for chromosome 15 (red; 3.5 μl; MetaSystems, Germany) and *SMAD6* gene-specific probe (*locus* 15q22.31; green; 0.5 μl; Agilent, USA); filled with HS to 5.0 μl;
- α-satellite for chromosomes: 15 (*locus* D15Z4; yellow = green + red), X (*locus* DXZ1; green) and Y (*locus* DYZ3; red) (Cytocell, UK); each probe 2.0 μl, filled with HS to 10.0 μl;
- α-satellite for chromosome 15 (*locus* D15Z4; yellow = green + red; each 2.0 μl) and band-specific for 21q22.13 (green) and 22q12 (red) (7.0 μl; Cytocell, UK); filled with HS to 10.0 μl;
- α-satellite for chromosome 15 (*locus* D15Z4; red; 1.5 μl; Cytocell, UK) and *SMAD6* gene-specific probe (*locus* 15q22.31; green; 0.5 μl; Agilent, USA), filled with HS to 5.0 μl.

Slide preparation was described previously^60^. All FISH experiments were performed according to the manufacturer’s standard protocol with minor modifications described elsewhere^23^,^60^. Briefly, only spermatozoa with unaffected tail after DTT treatment were selected for FISH analyzes. We adapted one FISH protocol (Cytocell, UK) to all FISH probes used in the study. Minor modifications concerned: lower volumes of probes used for centromere-specific probes, and simultaneous common denaturation of sperm DNA and mix of FISH probes on microscopic slide (2.5 min., 75 °C). Denaturation time for mixes containing *SMAD6* gene-specific probe was extended to 4 min. in 78 °C.

#### Microscopy and data analysis

Staining results were scored using light (for AB) and fluorescent microscopes (for CMA3 and FISH) (Zeiss D1 AxioImager) with an oil immersed 100× objective fitted with a proper filter set (FITC/SpO/DEAC/Triple/DAPI). The images were acquired using a CCD camera and analyzed with CellB (Olympus) or ISIS (MetaSystems, Germany) software. For meiotic segregation, 3,400 sperm cells of the sSMC carrier were evaluated. To investigate the aneuploidy level, at least 5,000 sperm cells were evaluated for each male (sSMC carrier; control males, n = 7; RF-group, n = 7) and chromosome. When two FISH signals in one colour were observed in a sperm cell, the criterion of the space between them (=minimum the size of the signal) was applied. The efficiency of FISH was estimated at 99%. For confocal analysis, a Nikon A1Rsi microscope (Japan) equipped with an appropriate range of lasers (405–641 nm) was used, followed by further image analyzes with Imaris software (Bitplane, Switzerland). To obtain 3D images, a series of image stacks were acquired for each spermatozoa (7 per μm of the nucleus depth along to the *z*-axis; an example of these stacks is presented in Suppl. Fig. S6).

### Centromere positioning within human spermatozoa

#### Radial positioning (2D)

**Figure 5:**
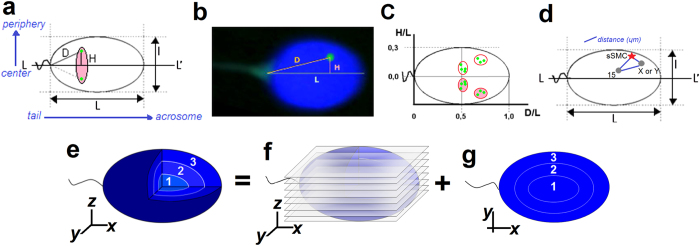
A schematic model of the radial and confocal spatial analyzes. (**a**) Schematic representation of the radial measurement method of centromere localization, according to the model of Zalenskaya and Zalensky^19^, including (**b**) representative FISH image. Green point: centromere; D and H: distances from centromere; L and l: long and short axes of sperm nucleus; pink area: mirror image of the centromeres’ position. Results depicted in a coordinate system as the normalized means D/L ± SE for OX axis and H/L ± SE for OY axis with (**c**) hypothetically marked chromocenter areas (aggregations of centromeres), including their mirror images. (**d**) Scheme of distance measurement (μm) method between FISH signals within sperm nucleus. (**e–g**) Schematic model of confocal sperm nucleus divided into three concentric shells (**e**) according to the depth of the nucleus: 1 (‘cen’) the most inner/central shell, 2 (‘int’) intermediate area, 3 (‘per’) peripheral area near the nuclear lamina. FISH signals were assigned to a particular shell after analysis of the *z*-axis stacks (**f**); (each shell consisted of 1/3 of the total number of stacks obtained for each sperm nucleus) and analysis of the *xy*-axes view (**g**) of the sperm nucleus.

The localization of centromeres of chromosomes 15, 18, X, Y and sSMC, as well as of *SMAD6* gene *locus* (15q22.31), was estimated with the radial evaluation technique, according to a method previously described^19^ and presented briefly in Fig. 5a–c. The following parameters were measured: L: length of the long axis (from tail attachment point to acrosome); l: short axis (at the widest part of the nucleus); and L/l: the ellipsoidal shape determining the decondensation ratio of the nucleus. The value of D/L indicated the location of the chromosome centromere with respect to the length of the sperm nucleus (the ‘tail-acrosome’ criterion; D: distance between the FISH signal and the sperm tail attachment). The H/L value indicated the location of the chromosome centromere in the depth of the nucleus (the ‘center-periphery’ criterion; H: distance between FISH signal and the L axis). The results were depicted on a coordinate system as the mean D/L ± SE for the OX axis and H/L ± SE for the OY axis. It is known that, on a microscopic slide, spermatozoa can obtain only two mirror positions, like a flipping coin. For each chromosome, about 100 FISH signals were analyzed, both for sperm cells with (sSMC^+^) for cells without the sSMC chromosome (sSMC^−^). A hierarchal Ward cluster analysis was also performed to check the aggregated localization of the chromosomes in the sSMC^+^ spermatozoa, as compared to the sSMC^−^ cells.

Distance was measured between selected pairs of the centromeres (in sSMC^+^ spermatozoa: sSMC vs. 15, X, Y and 15 vs. X, Y; in sSMC^−^: 15 vs. X, Y) for 100 sperm cells, in each case using ISIS software (MetaSystems, Germany) with the measurement tool option (Fig. 5d). To avoid nuclear swelling, the measured distances were divided by the L/l values (normalization). The results are shown as means ± SD (μm).

#### Confocal positioning (3D)

Confocal analysis was performed to determine the localization of sSMC and to compare the topology of chromosome 15 centromere in sSMC^+^ vs. sSMC^−^ spermatozoa. The distinction between the centromere of chromosome 15 and sSMC was possible because of the different size of the FISH signal – the sSMC signal being at least twice as small. To better visualize the chromosomes, a combination of centromere-15-specific FISH probe and gene-specific (*SMAD6*) probe was applied. The sperm cell nucleus was divided into three equally sized concentric shells by depth: from the innermost part of the nucleus (shell no. 1, ‘cen’), through an intermediate shell (no. 2, ‘int’), to the peripheral shell (no. 3, ‘per’) (Fig. 5e). A FISH signal was assigned to particular shell after analyzing the *z*-axis stacks (each shell consisted of one third of the total number of stacks obtained for each sperm nucleus; Fig. 5f) and the *xy*-axes view of the sperm nucleus (Fig. 5g, Suppl. Fig. S6). The frequency of FISH signals in each shell was estimated for 40 cells in sSMC^+^ and sSMC^−^ spermatozoa.

### Statistical analysis

The statistical significance between the mean values for the sperm aneuploidy level and sperm chromatin deprotamination was determined using χ^2^ and one-sample *t*-tests. For comparison of the normalized distances between centromeres, an unpaired *t*-test was performed. To check the clustered localization of the evaluated centromeres, a hierarchal Ward cluster analysis was carried out. For confocal positioning, a Fisher exact test was applied. All these tests were determined at the significance level of α = 0.05 using OriginLab (v. 8.5) and GraphPad Prism (v.5) software. For statistical analysis of the radial topology results, one-way analysis of variance (ANOVA; OriginLab) was used. The significance level of α = 0.01 for this test was more adequate for the biological significance of the observed alterations.

## Additional Information

**How to cite this article**: Olszewska, M. *et al.* Genetic dosage and position effect of small supernumerary marker chromosome (sSMC) in human sperm nuclei in infertile male patient. *Sci. Rep.* **5**, 17408; doi: 10.1038/srep17408 (2015).

## Supplementary Material

Supplementary Figures and Tables

Supplementary Video S4

Supplementary Video S5

## Figures and Tables

**Table 1:**
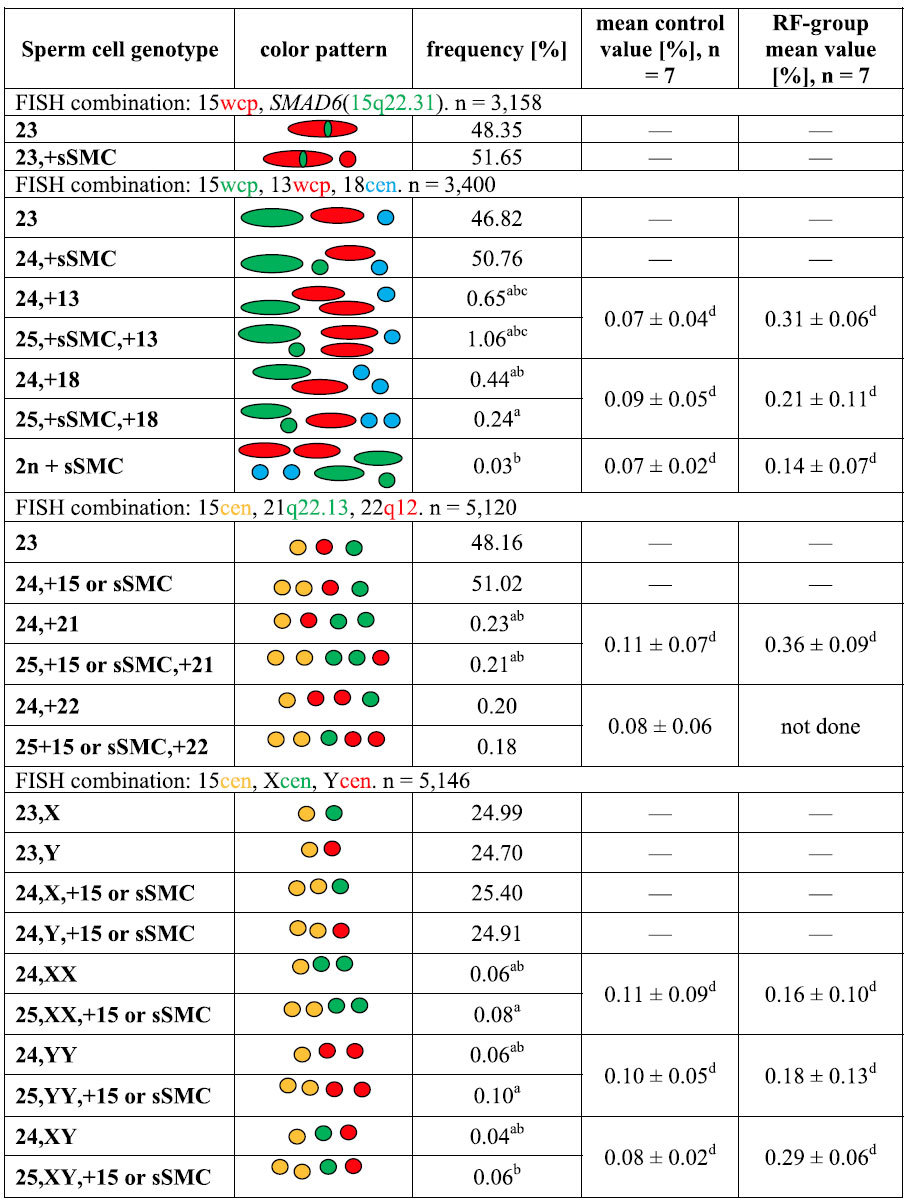
Meiotic segregation pattern and aneuploidy level of selected chromosomes in sperm cells from patient with 47,XY,+der(15)(pter->p11.2::q11.1->q11.2::p11.2->pter)mat karyotype.

The results obtained for the sSMC carrier [% of spermatozoa with proper genotype] were compared to two control groups of males: (i) healthy, fertile donors (mean control group) and (ii) males with reproductive failures (RF) and normal karyotype 46,XY (RF-group). sSMC: small supernumerary marker chromosome; cen: centromerespecific FISH probe; wcp: whole chromosome painting FISH probe. Mean values depicted as frequencies ½ SD. Statistical significance (asterisks; P < 0.05) according to mean control value^a^, RF-control value^b^, between sSMC^+^ and sSMC^-^ gametes^c^, or between the two control groups^d^ after one-sample t-test assay at the a = 0.05 significance level. Aneuploidy criteria: spermatozoa presenting basic colour pattern of FISH signals (listed in the Table), classified regarding one colour signal for each probe, were considered as normal; spermatozoa containing any additional colour signal, were considered as carrying sSMC and/or the other chromosome analyzed. Detailed colour patterns for observed aneuploidies are presented in the second column of the Table).

**Table 2:** Radial localization of centromeres of chromosomes: 15, 18, X, Y, sSMC and *SMAD6* gene locus (15q22.31) in sperm cell nuclei of a marker chromosome carrier karyotyped as 47,XY,+der(15)(pter->p11.2::q11.1->q11.2::p11.2->pter)mat.

sSMC presence	CHR	Mean D/L	SE	p value	Mean H/L	SE	P value
−	15	0.549	0.021	ns (0.034)	0.149	0.012	ns (0.028)
+		0.608	0.020		0.113	0.013	
−	18	0.543	0.018	ns (0.029)	0.165	0.010	ns (0.094)
+		0.484	0.021		0.141	0.009	
−	X	0.610	0.015	ns (0.643)	0.109	0.009	<0.0001
+		0.619	0.012		0.201	0.009	
−	Y	0.579	0.011	ns (0.023)	0.100	0.008	<0.0001
+		0.618	0.013		0.212	0.010	
−	*SMAD6*	0.557	0.017	ns (0.445)	0.123	0.007	ns (0.240)
+		0.575	0.016		0.134	0.009	
+	sSMC	0.641	0.016	ns (0.019)	0.198	0.005	<0.0001
+	15	0.608	0.020		0.113	0.013	
+	sSMC	0.641	0.016	ns (0.321)	0.198	0.005	ns (0.853)
+	X	0.619	0.012		0.201	0.009	
+	sSMC	0.641	0.016	ns (0.326)	0.198	0.005	ns (0.377)
+	Y	0.618	0.013		0.212	0.010	

‘+’ means spermatozoa bearing sSMC, while ‘−’ represents spermatozoa without sSMC. Mean D/L and H/L values: mean values obtained for 100 spermatozoa for each chromosome; SE: standard error. Value P < 0.01 indicated statistically significant difference; ns: no statistical significance (P > 0.01).
