# Supplementary material for: Genetic dosage and position effect of small supernumerary marker chromosome (sSMC) in human sperm nuclei in infertile male patient
Source: Sci Rep. 2015 Nov 30;5:17408. doi: 10.1038/srep17408 (PMC4663790; doi:10.1038/srep17408)
Supplement: Supplementary Figures and Tables [file srep17408-s1.doc]

**Supplementary files for the manuscript:**

**Genetic dosage and position effect of small supernumerary marker chromosome (sSMC) in human sperm nuclei in infertile male patient**

Marta Olszewska^1^, Elzbieta Wanowska^1^, Archana Kishore^2^, Nataliya Huleyuk^3^, Andrew P. Georgiadis^2^, Alexander N. Yatsenko^2^, Mariya Mikula^3^, Danuta Zastavna^3^, Ewa Wiland^1^, Maciej Kurpisz^1*^

^1^ Institute of Human Genetics, Polish Academy of Sciences, Department of Reproductive Biology and Stem Cells, Strzeszynska 32, 60-479 Poznan, Poland;

^2^ Department of Obstetrics, Gynecology and Reproductive Sciences, University of Pittsburgh School of Medicine, Pittsburgh 15213, PA, USA

^3^ Institute of Hereditary Pathology, Ukrainian Academy of Medical Sciences, Lysenko Str. 31a, 79000 Lviv, Ukraine

*corresponding author: Maciej Kurpisz, PhD, MD

Institute of Human Genetics Polish Academy of Sciences

Department of Reproductive Biology and Stem Cells

Strzeszynska 32, 60-479 Poznan, Poland

Phone: +4861 6579 202/212, Fax: +4861 8233 235

e-mail: [kurpimac@man.poznan.pl](mailto:kurpimac@man.poznan.pl)


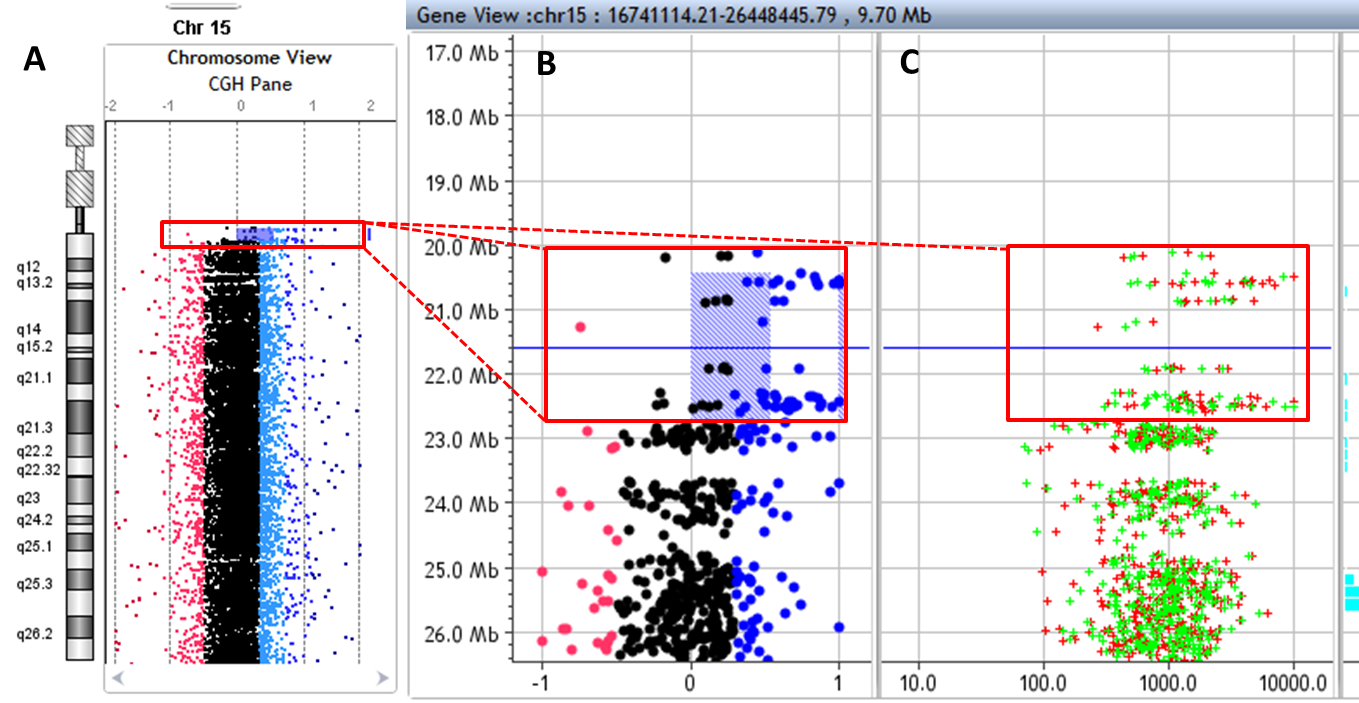
**Supplementary Figure S1.** Array CGH profile showing the 15q11.1-q11.2 amplification. The shaded blue area indicates amplification of ~2.3Mb size with a log ratio of 0.547. The genes included in the region are *HERC2P3, GOLGA6L6, GOLGA8C, BCL8, POTEB, NF1P1, LOC646214, CXADRP2, LOC727924, OR4M2, OR4N4, OR4N3P, REREP3, GOLGA8DP, GOLGA6L1*.

| **#** | **Chr** | **Cyto-band** | **Start** | **Stop** | **#Probes** | **Amplification** | **Deletion** | **P value** | **Gene Names** | **Comments** |
| --- | --- | --- | --- | --- | --- | --- | --- | --- | --- | --- |
| 1 | chr4 | q13.2 | 69,387,056 | 69,483,277 | 12 | 0.780181 | 0 | 8.58E-16 | *UGT2B17, UGT2B15* | polymorphic |
| 2 | chr7 | p22.3 | 1,071,991 | 1,081,240 | 3 | 1.272417 | 0 | 5.42E-14 | *C7orf50* | intronic |
| 3 | chr7 | q34 | 141,766,883 | 141,792,094 | 6 | 0 | -0.8426 | 4.28E-13 | *MGAM* | polymorphic |
| 4 | chr8 | p11.22 | 39,222,367 | 39,392,534 | 31 | 0.390459 | 0 | 3.18E-12 | *ADAM5P, ADAM3A* | polymorphic |
| 5 | chr15 | q11.1- q11.2 | 20,432,851 | 22,756,709 | 63 | 0.546536 | 0 | NA | *HERC2P3, GOLGA6L6, GOLGA8C, BCL8, POTEB, NF1P1, LOC646214, CXADRP2, LOC727924, OR4M2, OR4N4, OR4N3P, REREP3, GOLGA8DP, GOLGA6L1* |  |

**Supplementary Table S2.** aCGH results showing all aberrations.

**Supplementary Table S3.** Genes present in the amplified region of chromosome 15 as detected by aCGH.

| **Gene name** | **Function/description** | **Human Expression (BioGPS)** |
| --- | --- | --- |
| *HERC2P3* | pseudogene | ubiquitous |
| *GOLGA6L6* | Golgi autoantigen | unknown |
| *GOLGA8C* | Golgi autoantigen, pseudogene | Testis elevated |
| *BCL8* | Pseudogene (renamed *NBEAP1*) | unknown |
| *POTEB* | Cancer/testis antigen family gene (may play a role in spermatogenesis) | Unknown (testis-specific in RNA-seq data from GeneCards) |
| *NF1P1* | pseudogene | unknown |
| *LOC646214* | pseudogene | unknown |
| *CXADRP2* | pseudogene | unknown |
| *LOC727924* | Noncoding RNA | unknown |
| *OR4M2* | Olfactory receptors | Unknown (testis only in RNA-seq data from GeneCards) |
| *OR4N4* | Olfactory receptors | Unknown (testis only in RNA-seq data from GeneCards) |
| *OR4N3P* | Olfactory receptors, pseudogene | unknown(testis only in RNA-seq data from GeneCards) |
| *REREP3* | pseudogene | unknown |
| *GOLGA8DP* | Golgi antigen, pseudogene | Testis elevated |
| *GOLGA6L1* | Golgi antigen | Unknown (testis elevated in RNA-seq data from GeneCards) |

**Supplementary Video S4.** Spermatozoa bearing sSMC. Red: centromere-specific probe for chromosome 15; green: *SMAD6* gene *locus* (15q22.31).

**Supplementary Video S5.** Spermatozoa without sSMC. Red: centromere-specific probe for chromosome 15; green: *SMAD6* gene *locus* (15q22.31).

**These two files are uploaded separately.**


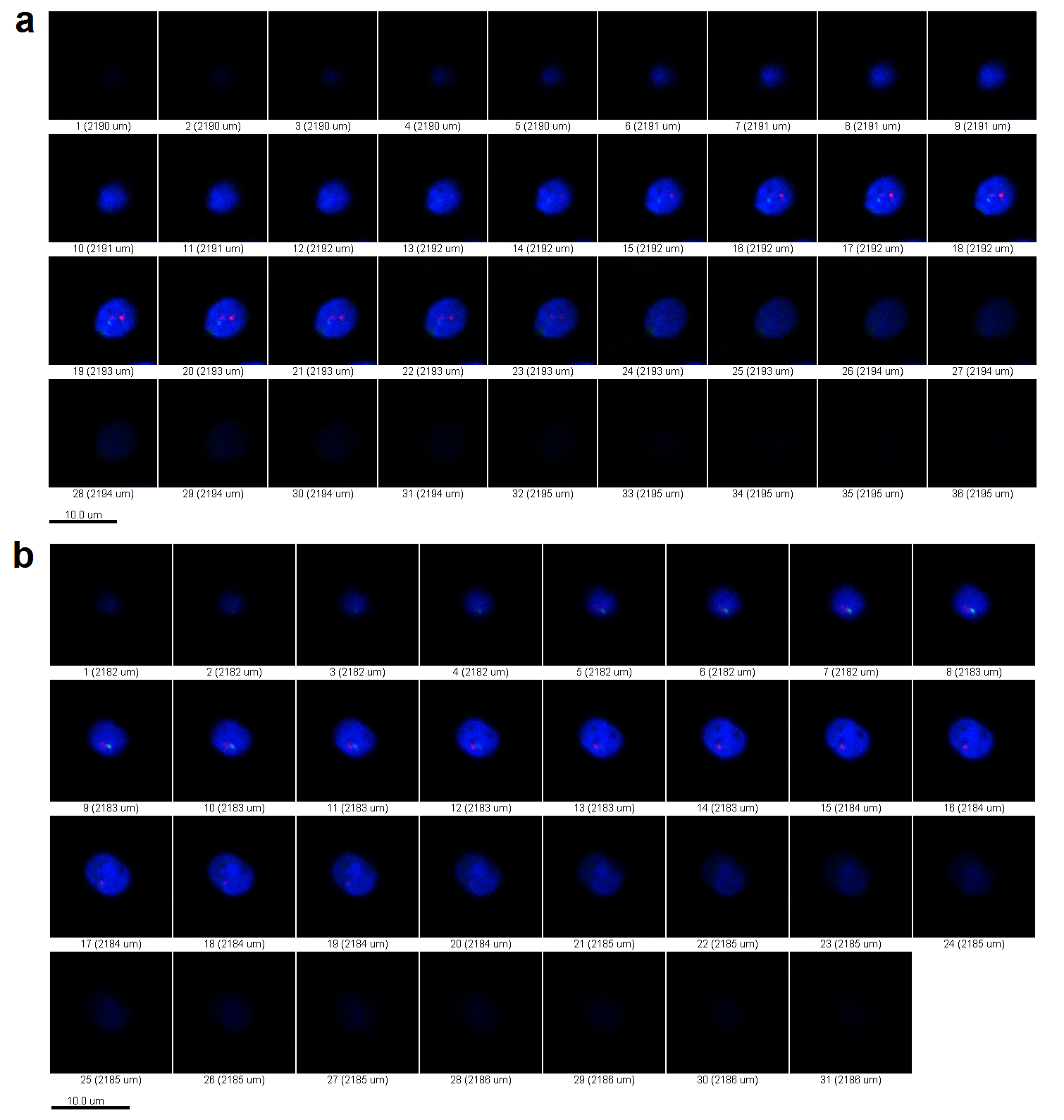
**Supplementary Figure S6.** Stack of pictures collected along the *z*-axis within confocal microscopy. For each spermatozoa, series of 7 stacks per µm of nucleus depth were acquired. Red: α-satellite probe for chromosome 15; green: *SMAD6* gene *locus* (15q22.31). **a.** sSMC^+^ spermatozoa with two red (15 and sSMC) and one green FISH signal (*SMAD6*). **b.** sSMC^-^ spermatozoa with one red and one green FISH signal (only chromosome 15).
